# Unlocking the power of short-chain fatty acids in ameliorating intestinal mucosal immunity: a new porcine nutritional approach

**DOI:** 10.3389/fcimb.2024.1449030

**Published:** 2024-09-02

**Authors:** Haoyang Liu, Hongde Lu, Yuxuan Wang, Chenyun Yu, Zhiyuan He, Hong Dong

**Affiliations:** ^1^ Beijing Key Laboratory of Traditional Chinese Veterinary Medicine, Beijing University of Agriculture, Beijing, China; ^2^ Beijing Engineering Research Center of Chinese Veterinary Medicine, Beijing University of Agriculture, Beijing, China

**Keywords:** porcine, short-chain fatty acids, gut microbiota, intestinal mucosal immunity, nutrition

## Abstract

Short-chain fatty acids (SCFAs), a subset of organic fatty acids with carbon chains ranging from one to six atoms in length, encompass acetate, propionate, and butyrate. These compounds are the endproducts of dietary fiber fermentation, primarily catalyzed by the glycolysis and pentose phosphate pathways within the gut microbiota. SCFAs act as pivotal energy substrates and signaling molecules in the realm of animal nutrition, exerting a profound influence on the intestinal, immune system, and intestinal barrier functions. Specifically, they contibute to 60-70% of the total energy requirements in ruminants and 10-25% in monogastric animals. SCFAs have demonstrated the capability to effectively modulate intestinal pH, optimize the absorption of mineral elements, and impede pathogen invasion. Moreover, they enhance the expression of proteins associated with intestinal tight junctions and stimulate mucus production, thereby refining intestinal tissue morphology and preserving the integrity of the intestinal structure. Notably, SCFAs also exert anti-inflammatory properties, mitigating inflammation within the intestinal epithelium and strengthening the intestinal barrier’s defensive capabilities. The present review endeavors to synthesize recent findings regarding the role of SCFAs as crucial signaling intermediaries between the metabolic activities of gut microbiota and the status of porcine cells. It also provides a comprehensive overview of the current literature on SCFAs’ impact on immune responses within the porcine intestinal mucosa.

## 1 Introduction

Short-chain fatty acids (SCFAs), integral metabolites of the gut microbiota, have garnered significant attention in the scientific community for their pivotal role in maintaining host health and immue function (Koh et al., 2016; O’Riordan et al., 2022; Zhang et al., 2023). The gut microbiota is acknowledged for its critical role in upholding host health and immune function, with SCFAs posited as key players in the sustenance of gut homeostasis and the balance of the immune system (Ratajczak et al., 2019). In this regard, considerable research endeavors have been dedicated to elucidating the myriad functions of SCFAs and evaluating their impact on intestinal health and immune regulation. Previous studies have predominantly concentrated on examining the influence of SCFAs on intestinal well-being and growth efficiency in pigs (Che et al., 2019; Zhao et al., 2022; Lan et al., 2023). These contributions have furnished the field with foundational insights into the multifaceted roles of SCFAs within the gastrointestinal milieu. Nonetheless, a robust and holistic understanding of the mechanisms by which SCFAs modulate mucosal immune responses remains an area that warrants further investigation. While previous reviews have predominantly leaned on *in vitro* studies and animal models, particularly murine, to extrapolate the effects of SCFAs on immune signaling pathways, such as NF-κB, Wnt/β-Catenin and MAPK (Li et al., 2020; McBrearty et al., 2021; Wang et al., 2023; Chen et al., 2024). The direct applicability of these findings to porcine mucosal immunity requires additional validation. Therefore, the aim of this review is to consolidate current knowledge regarding the regulatory functions of SCFAs in porcine mucosal immunity and to present novel perspectives that may guide future research endeavors in this domain.

Piglets lack passive immunity, a condition that is unique to them due to the filtration function of the sow’s uteroplacental epithelial villi, which restricts the passage of immunoglobulins and other macromolecular substances (Vodolazska et al., 2023). Unlike humans, the differentiation of T and B lymphocytes in piglets and their commencement of immune functions begin approximately three weeks postnatal, with full maturation achieved by eight weeks of age (Suganuma et al., 1986; Sinkora et al., 2005). In piglets, pulmonary macrophages reach adult functionality levels around two weeks of age, while those in the bloodstream differentiate and mature between 3 to 7 days postnatal (Rothlein et al., 1981). Despite a surge in the population of porcine neutrophils during the initial weeks of life, their chemotactic ability remains relatively diminished (Mccauley and Hartmann, 1984). Natural killer cells, initially quiescent in newborn piglets, undergo a maturation process that spans approximately 2 to 3 weeks (Harayama et al., 2022).

Piglets are born agammaglobulinemic, exhibiting limited cellular immune capabilities. They possess a reduced count of peripheral lymphoid cells, underdeveloped lymphoid tissues, and are predominantly lacking in effector and memory T cells (Leidenberger et al., 2017). Due to these factors, securing adequate passive immunity is crucial for piglets, which requires the ingestion of colostrum within the first 36 hours of life (Levast et al., 2014). Porcine colostrum is rich in immunoglobulins (Ig), comprising roughly 80% IgG and 20% IgA. In addition to Ig, it contains a variety of other bioactive components, including growth factors, cytokines, enzymes, antimicrobial substances, and cellular components such as neutrophils and lymphocytes. These elements are efficiently absorbed by the neonatal gastrointestinal tract through the act of swallowing, thereby conferring substantial passive immune protection (Quesnel, 2011). The intestinal epithelial barrier in newborn piglets remains permeable to antibodies and other large molecules until the process of gut closure, which facilitates the absorption of Igs (Matson et al., 2010; Choudhury et al., 2021). Maternal immunization can endow newborn piglets with passive immunity against a range of infections; however, it may concurrently impede the development of vaccine-induced immunity (Pravieux et al., 2007; Yuan et al., 2023). Consequently, the immunity established at mucosal surfaces in sows is conveyed to nursing piglets through colostrum feeding, a phenomenon known as lactogenic immunity. This mechanism is vital for providing immediate protection against enteric pathogens in piglets (Langel et al., 2016).

Short-chain fatty acids (SCFAs), also known as volatile fatty acids, are characterized by carbon chains of fewer than six atoms and primarily consist of acetate, propionate, and butyrate (Zhang et al., 2024). These SCFAs are the predominant end products of microbial fermentation in the colon and serve as the principal anions within the hindgut of mammals (Nogal et al., 2021). In the absence of dietary supplementation, SCFA concentrations in the hindgut typically fall within the range of 58-69 mmol/kg dry matter (DM). However, a diet rich in fiber can elevate endogenous organic acid production to levels of 84-98 mmol/kg DM (Rossi et al., 2010). The rate of production and distribution of SCFAs largely depends on the diversity and population of the colonic gut microbiota, as well as the nature of the substrate and the transit time within the intestine (Wong et al., 2006). The genera primarily responsible for SCFAs synthesis include *Eubacterium*, *Roseburia*, *Clostridium anaerobes*, *Streptococcus*, *Bacteroides* and *Bifidobacterium* (Layden et al., 2013; Ziegler et al., 2016; McNabney and Henagan, 2017; Mehta et al., 2022). Acetate is predominantly generated through fermentation by *Bifidobacterium* and *Lactobacillus Heprevotella*. While, Propionate is mainly produced via the fermentation of Bacteroides and members of the phylum Firmicutes. In contrast, butyrate is predominantly the result of fermentation by Firmicutes, particularly species within the *Verrucomicrobiaceae* and *Lachnospiraceae* families (Louis and Flint, 2017; Bach Knudsen et al., 2018; Rowland et al., 2018; Markowiak-Kopeć and Śliżewska, 2020; Asadpoor et al., 2021). Further investigation is warranted to elucidate the complex interplay between dietary intake, the diversity and functionality of gut microbiota, and their collective impact on porcine intestinal health.

As indispensable energy sources, SCFAs exert pivotal influences on various physiological processes of the host. They significantly impact nutritional status, immune system development, cellular metabolism, intestinal barrier function, and motility (Nakatani et al., 2018; Metzler-Zebeli et al., 2021). Among SCFAs, the production of butyrate by specific gut microbiota is instrumental in promoting the growth and development of both the large and small intestines (Zhu et al., 2021). Additionally, butyrate stimulates the expression of tight junction proteins within the intestinal mucosa, which is crucial for promoting wound healing and maintaining the integrity of the intestinal barrier (Chen et al., 2018). In addition to these roles, SCFAs exhibit broad-spectrum antimicrobial properties, enhancing the host’s defense mechanisms against a range of pathogens (Fernández-Rubio et al., 2009; Xiong et al., 2016). Furthermore, they modulate both innate and adaptive immune responses, influencing leukocyte recruitment and mitigating intestinal inflammation (Li et al., 2017; Ouyang et al., 2024).

The primary objective of this study is to conduct a comprehensive review of the regulatory role of short-chain fatty acids (SCFAs) in mucosal immunity within the porcine model, thereby offering novel insights into this area of research. Our investigation will specifically address several key questions: What is the impact of SCFAs on mucosal immune mechanisms in pigs? How do these compounds contribute to the activation of immune cells and the regulation of inflammation? Additionally, we will examine their influence on intestinal barrier function and antimicrobial defense in pigs. Our review will also examine the potential regulatory role of SCFAs in gut immune recovery and their interactions with other immune-modulating agents. By synthesizing the latest research findings, we aim to provide a thorough understanding of the multifaceted functions of SCFAs in modulating mucosal immunity in pigs. Initially, we will focus on the role of SCFAs in pigs as a significant animal model, establishing a foundation that could be extrapolated to clinical applications. Subsequently, we will provide a summary of the most recent research, elucidating the intricate mechanisms through which SCFAs interact with mucosal immunity in pigs, thereby enhancing our grasp of immune regulation. Moreover, we will delve into the cross-talk between SCFAs and other factors known to modulate the immune system, with the goal of offering innovative perspectives that could inform future research directions and therapeutic strategies.

## 2 Regulation of the gastrointestinal barrier function by SCFAs

**Figure 1:**
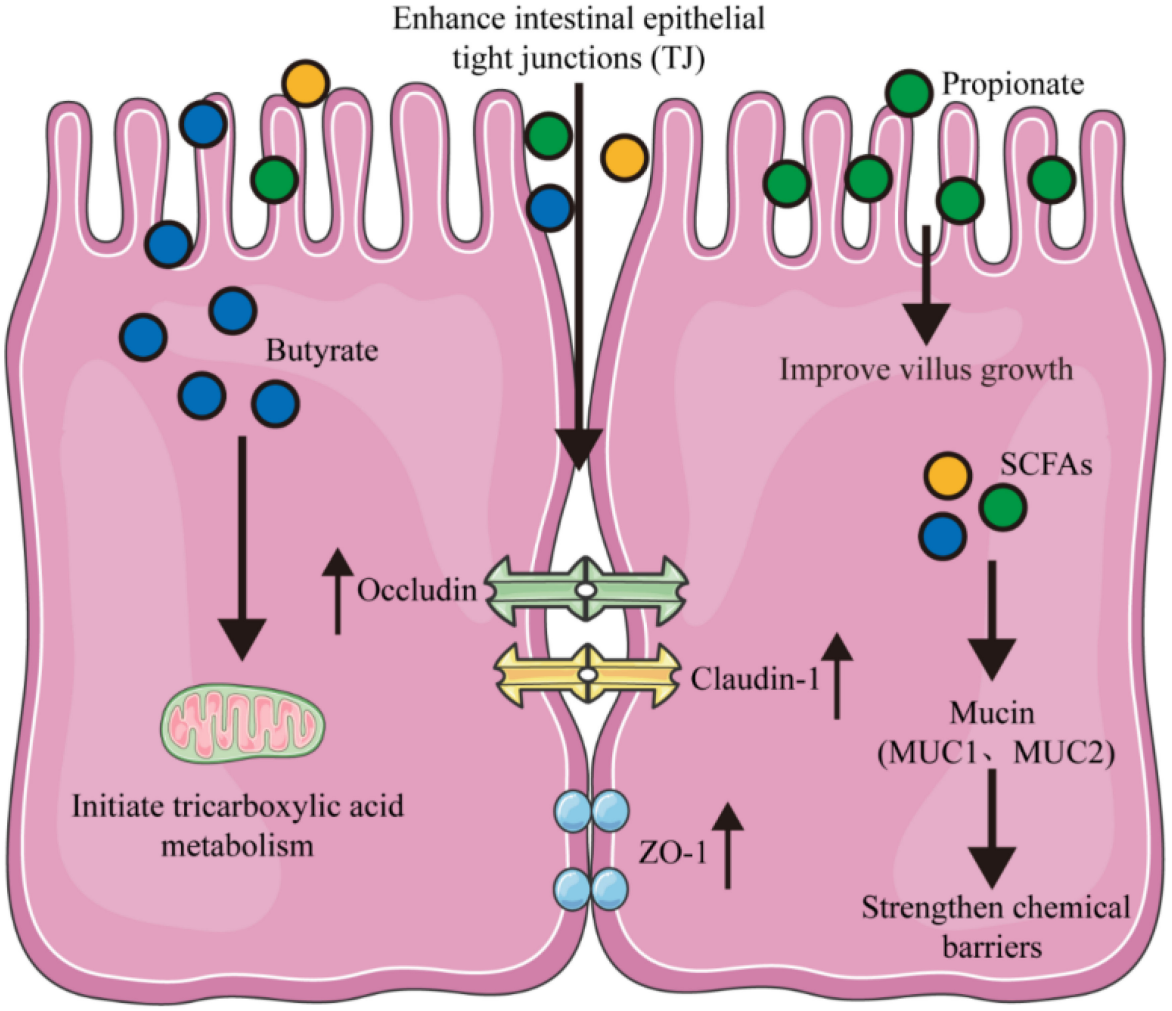
Maintenance of intestinal barrier function via SCFAs. SCFAs coordinate the expression of tight junction proteins (ZO-1, Occludin, and Claudin-1) to enhance intestinal barrier function. Intracellular SCFAs improve chemical barriers via mucins (MUC1, MUC2). Dietary propionate and butyrate promote the growth of villi in intestinal epithelial cells and activate tricarboxylic acid metabolism by entering the mitochondria.

The intestinal barrier serves a dual role, not only as the central hub for nutrient digestion and absorption but also as the frontline defense mechanism against the incursion of pathogenic microorganisms and hazardous substances. This dual function is pivotal in preserving the integrity of normal health status (Harikrishnan, 2019). Among its various components, short-chain fatty acids (SCFA) are particularly important for sustaining the integrity of the tight junctions (TJ) between intestinal epithelial cells (**Figure 1**). By doing so, they help reduce damage caused by pathogens and facilitate the recovery of the intestinal mucosal barrier function (Ji et al., 2022; Yue et al., 2022).

Previous studies have demonstrated that SCFAs undergo hepatic metabolism, where they are converted into ketone bodies, such as acetoacetate and β-hydroxybutyrate, as well as glutamate. These metabolites serve as essential fuels for oxidative metabolism in the small intestine (Zhao et al., 2024). SCFAs also stimulate the production of nerve signals that connect the autonomic and central nervous systems, thereby influencing overall metabolism (Zhang et al., 2019). Moreover, SCFAs promote the secretion of hormones and growth factors by intestinal epithelial cells, which enhances intestinal development. Chen et al. demonstrated that the dietary supplementation of xylo-oligosaccharides significantly increased the production of short-chain fatty acids, particularly butyrate and propionate (Chen et al., 2021). This enhancement induced a series of physiological responses in swine, including alterations in cell proliferation, a reconfiguration of energy metabolism, and an enhancement of the intestinal barrier. Furthermore, it modulated the intestinal immune system, underscoring the potential of xylo-oligosaccharides as a dietary intervention for gut health (Chen et al., 2021; Li et al., 2022). Butyrate, in particular, contributes 60%-70% of the energy source for colonic epithelial cells. Once absorbed by intestinal epithelial cells, butyrate penetrates mitochondria to initiate tricarboxylic acid metabolism (Zhang et al., 2019; Lee et al., 2022). SCFAs have also been found to reduce intestinal permeability and improve the integrity of intestinal epithelium by upregulating proteins such as zonula occludens-1 and tight junction protein occludin-5 (Bendriss et al., 2023). In a hyperuricemia model using C57BL/6J mice, SCFAs produced by the intestinal microbiota were found to upregulate mRNA and protein levels related to tight junctions, leading to an increase in tight junctions between intestinal epithelial cells and restoring the intestinal mechanical barrier function (Guo et al., 2021). When administering gastric infusions to weaned piglets, SCFAs increase serum and digestive fluid concentrations, enhance the distribution of SCFA receptors in porcine intestinal tissue, and upregulate genes related to intestinal development (Diao et al., 2019). Additionally, SCFA supplementation decreases the abundance of proapoptotic genes and proteins, promoting piglet growth and reducing diarrhea incidence (Huang et al., 2015). In another study with early-weaned piglets, dietary supplementation with sodium butyrate (2000 mg/kg) resulted in a significant increase in mRNA expression levels of ZO-1, occludin, and claudin-3, as well as protein expression levels of occludin and claudin-3 (Feng et al., 2018). This supplementation reduced intestinal permeability, alleviated diarrhea, and improved porcine growth performance. Furthermore, when microbial butyrate formation is low or absent, adding butyrate to total parenteral nutrition (TPN) formulas for neonatal piglets has been shown to stimulate tissue regeneration after jejunoileal resection (Bartholome et al., 2004). In summary, SCFAs primarily regulate the proliferation of intestinal cells and metabolic processes, highlighting their crucial role in intestianl health and development.

During early life, the concentrations of short-chain fatty acids (SCFAs) in piglets increase, with acetate levels decreasing while butyrate and propionate levels rise significantly (Qi et al., 2020). Dietary supplementation with sodium butyrate has been shown to enhance average daily gain (ADG) and improve the feed conversion ratio in weaned piglets. Additionally, it reduces serum malondialdehyde levels and the incidence of diarrhea by modulating intestinal permeability and the structure of the intestinal microbiota (Huang et al., 2015). Butyrate specifically has been found to bolster intestinal barrier function. It does this by increasing the mRNA expression levels of tight junction proteins and facilitating their reassembly, as well as by elevating transepithelial electrical resistance (TER) in Caco-2 and IPEC-J2 cells (Sekirov et al., 2010; Ma et al., 2012; Diao et al., 2019). Propionate, on the other hand, has been shown to increase villus height and the villus height-to-crypt depth ratio in the jejunum of finishing pigs (Zhang et al., 2019). Therefore, further research comparing the effects of different sources and forms of SCFAs on piglets is essential. This will help to confirm their potential to enhance gastrointestinal barrier function.

## 3 The influence on the gut microbiota by SCFAs

The diversity and relative abundance of gut microbiota vary across different intestinal sites. The total number of bacterial cell count escalates from the ileum to the colon, peaking in the distal intestine of swine. Moreover, bacterial composition is site-specific along the the GI tract (Sekirov et al., 2010; Biagi et al., 2012). Gut microbiota is influenced by both external factors, such as diet and antibiotics, and internal metabolic processes. Short-chain fatty acids (SCFAs) play a pivotal role in modulating the intestinal environment by reducing pH and redox potential (Zheng et al., 2017), thereby fostering the growth of beneficial probiotics and suppressing the proliferation of pathogenic and commensal bacteria (Lu et al., 2022; Kang et al., 2024). SCFAs also enhance intestinal colonization resistance, which in turn can alter the gut microbiota’s structure (Duan et al., 2021). *Bifidobacterium* and *Lactobacillus* not only stimulate the secretion of intestinal mucin proteins, but also impede the adhesion of pathogens, such as *Escherichia coli* and *Clostridium perfringens*, to mucus and epithelial cells by acidifying the environment through SCFA production (Rauch et al., 2022). Previous research has identified *Clostridium butyricum* and *Enterococcus faecalis* as crucial probiotics, beneficial for the healthy development of weaned piglets. Dietary supplementation with these probiotics can enhance intestinal growth performance and protect hepatocytes and intestinal villi (Wang et al., 2019). Plant-derived natural analogs play the same role by affecting gut bacterial abundance, Astragalus and Ginseng polysaccharides regulate host immune functioning by activating the TLR4-mediated MyD88-dependent signaling pathway and increase volatile fatty acids (VFAs) in the colonic contents, improvement of intestinal morphology (Yang et al., 2019). This enrichment of colonic microbial populations and diversity is observed in weaned piglets. Therefore, the addition of natural analogs to the diet is also a way to improve the immunity of piglets to maintain normal intestinal morphology. It is well established that SCFAs provide essential energy for bacterial communities, lower gut lumen pH, and contribute to an acidic environment (Yang et al., 2024). *In vitro* tests have demonstrated that propionic acid can inhibit *Staphylococcus aureus* and inactivate *Salmonella* and *Escherichia coli* at pH 5 (Alva-Murillo et al., 2012). Butyric acid promotes the adhesion of beneficial probiotics such as *Bifidobacteria* and *Lactobacilli*, while reducing the adhesion of *Escherichia coli* (Cai et al., 2022). Consistent with *in vitro* findings, *in vivo* studies have have shown that high concentrations of SCFAs can reduce the presence of conditional pathogens, like *Salmonella* and *Escherichia coli* in the porcine intestine (Zhu et al., 2021). The dietary inclusion of sodium butyrate has been shown to enhance the proliferation of *Lactobacilli* in the porcine intestinal tract, while decreasing *Salmonella* and *Escherichia coli*, particularly in the duodenum, thus significantly impacting the overall microbiota balance (Gálfi and Bokori, 1990). The addition of berberine (BBR) and ellagic acid (EA) to the diet of weaned piglets can improve growth performance and gut health by modifying gut microbiota composition and increasing propionic and butyric acid concentrations (Qin et al., 2023). Furthermore, incorporating SCFAs and medium-chain fatty acids (MCFA) along with natural plant extracts (PHY) into the diet has been shown to positively influence diarrhea prevention and growth performance in weaned piglets, suggesting effective control of pathogenic *Escherichia coli* in weaned piglets (Caprarulo et al., 2023). Importantly, SCFAs can restructure the gut microbiota, and the diversity and abundance of the gut microbiota can reciprocally affect SCFA production. For instance, metabolites such as acetic and lactic acids, produced by the proliferation of *Bifidobacteria* and *Lactobacilli* in the gut, serve as energy sources for certain other bacteria, enabling them to proliferate and produce butyric acid (Duysburgh et al., 2021).

As key metabolites of the gut microbiota, short-chain fatty acids (SCFAs) exhibit considerable potential as feed additives to improve the species composition and community structure of the porcine gut microbiota. However, there remains a significant amount of debate regarding their impact on the early colonization stages of gut microbiota.

## 4 Modulation of the porcine immune system by SCFAs

Short-chain fatty acids (SCFAs) exert a profound influence on the porcine immune system, modulating crucial aspects such as mucosal immunity, adaptive immune responses, and the immune-protective attributes of milk. A substantial body of literature has consistently highlighted the pivotal role of SCFAs in these immunomodulatory processes. With an evolving comprehension of their underlying mechanisms, it is imperative to dissect the multi-dimensional effects of SCFAs. This discourse will delve into the intricate influence of SCFAs on the immune system of pigs, focusing on the mucosal immune response, the protective role of milk in immune defense, and the adaptive immune system.

Short-chain fatty acids (SCFAs) are pivotal in modulating the mucosal immune function in pigs. They can achieve this by influencing mucin expression in the gut (Ma et al., 2022). Also preserve the integrity of the intestinal mucosa and bolster the immune response by regulating key mucosal immune cells, including dendritic cells and macrophages (Luu et al., 2020), as well as the impact on the immunometabolism and epigenetic status of regulatory lymphocytes to accomplish this. A study by Walia K et al. demonstrated that the dietary inclusion of sodium butyrate during the late fattening stage in pigs not only reduced the prevalence and serum positivity of *Salmonella* but also promoted growth (Walia et al., 2016). This finding implies that SCFAs may positively influence the pig’s intestinal mucosal barrier function and immune response, potentially decreasing susceptibility to pathogen infections and bolstering resistance to external environmental challenges. Research by Lin et al. revealed that the supplementation of lactic acid bacteria-fermented formula milk as an intestinal regulator led to several beneficial outcomes in weaned piglets (Lin et al., 2023). Specifically, compared with the CON group, piglets in the LFM group had significantly higher ileal lactate levels, which may be related to the lactate producing bacteria or the compound acidifier in LFM. It is not only beneficial to intestinal health, but also produces more SCFAs in the presence of lactic acid-utilizing bacteria (e.g. Acetitomaculum). Additionally, the supplementation resulted in a significant increase in the number of goblet cells within the crypts. These findings suggest that lactic acid bacteria-fermented formula milk can enhance intestinal homeostasis and mitigate the weaning stress experienced by piglets.

Short-chain fatty acids (SCFAs) have been extensively studied for their influence on milk immune protection in lactating pigs. Sows convey vital immune factors to their piglets through milk, safeguarding them against pathogenic microorganisms (Dos Santos Bersot et al., 2019). SCFAs are known to modulate these immune components, including immunoglobulins present in sow’s milk. A feeding trial by Galfi P et al. revealed that diets supplemented with sodium butyrate positively affected piglet growth performance (Gálfi and Bokori, 1990). This suggests that the SCFA butyrate might enhance milk immune protection either by altering the immune factor content in the milk or by modulating the intestinal microbiota. Further research has confirmed that SCFAs significantly regulate mucosal immune function, adaptive immune responses, and milk immune protection in pigs. These findings offer crucial insights into the intricate regulatory mechanisms of the pig immune system, emphasizing the importance of SCFAs in both direct and indirect immune system modulation.

Recent studies have underscored the significant regulatory role of short-chain fatty acids (SCFAs) in shaping the adaptive immune responses in pigs. SCFAs modulate inflammatory responses within the intestinal tract by influencing the activity of immune cells (Yang et al., 2024). Specifically, they inhibit the activation of pro-inflammatory T cells and stimulate the differentiation of immune regulatory T cells, known as Tregs (Qiu et al., 2022). This dual action helps maintain a balanced immune response. Moreover, SCFAs directly influence antibody production and the functionality of B cells (Kim et al., 2020), which are crucial for adaptive immunity. Smith et al. discovered that microbial metabolites, including SCFAs, are instrumental in maintaining the homeostasis of colonic Tregs (Smith et al., 2013). This regulation can significantly impact pigs’ immune responses and their ability to resist diseases. Furthermore, a review by Anshory et al. highlighted the complex nature of butyrate’s role in immune-related diseases (Anshory et al., 2023). While beneficial for intestinal health, butyrate may also carry other potential risks, For example, Kaiko et al. showed that butyrate administration leads to a reversal of autophagy in colon cells. As a result, colon cells preferentially break down butyrate, thereby avoiding harm to stem cells (Kaiko et al., 2016). This duality underscores the importance of carefully considering the regulatory effects of SCFAs on the immune system in practical applications.

## 5 Alleviation of intestinal inflammation by SCFAs

During the inflammatory response, endothelial cells facilitate the expression of cell adhesion molecules on activated immunocytes, including E-selectin, ICAM-1, and VCAM-1 (Birch et al., 2023). This process results in the adhesion of leukocytes to the vascular endothelium and their subsequent migration to specific tissues, thereby amplifying the inflammatory response. Previous studies have indicated that short-chain fatty acids (SCFAs) can mitigate intestinal inflammation by modulating both innate and adaptive immune responses and by influencing the leukocyte recruitment process (Li et al., 2017; Ouyang et al., 2024). SCFAs stimulate the expression of the NF-κB transcription factor in intestinal epithelial cells through Toll-like receptors (TLRs), which in turn promotes the secretion of TNF-α and diminishes the expression of IL-8 and MCP-1 (Kopczyńska and Kowalczyk, 2024; Ma et al., 2024). Furthermore, SCFAs can enhance the expression of selective adhesion molecules on neutrophil surfaces (L-selectin) and neutrophil chemoattractants (CINC-2αβ), which facilitates the release and migration of neutrophils to sites of inflammation (Yang et al., 2018). Additionally, SCFAs promote the expression of genes involved in plasma cell differentiation in B cells, accelerating their maturation into plasma cells (Shinde et al., 2020). Plasma cells are instrumental in increasing the secretion of various antibodies in the intestinal mucosa, such as secretory IgA, which plays a pivotal role in the local regulation of intestinal microorganisms and in controlling inflammation (Garcia-Castillo et al., 2019).

Many studies have shown that SCFAs can inhibit the production of pro-inflammatory mediators, such as TNF-α, IL-6 and NO, induced by LPS and many cytokines. They can promote the release of anti-inflammatory cellular factor IL-10, and reduce the expression level of IL-10 in monocytes (Vinolo et al., 2011). Besides, SCFAs down-regulate the PPARg and NF-κB transcription factors, thus inhibiting the expressions of ICAM-1 and VCAM-1. They can also block the secretion of proinflammatory factors, such as IL-2, IL-6 and TNF-α (Li et al., 2020; Zhou et al., 2022). Zhang et al. conducted an in-depth investigation into the modulatory effects of propionate on inflammatory gene expression (Zhang et al., 2018). Utilizing a porcine cecal fistulae model, they instilled sodium propionate directly into the cecum to precisely assess its impact on local inflammatory responses. NF-κB and IL-18 expression was found to be upregulated after propionate infusion. Moreover, the abundance of *Bacteroidetes* increased and the abundance of *Firmicutes* decreased. This study aimed to elucidate the role of short-chain fatty acid propionates in the regulation of colonic microbiota and inflammatory cytokines.

**Figure 2:**
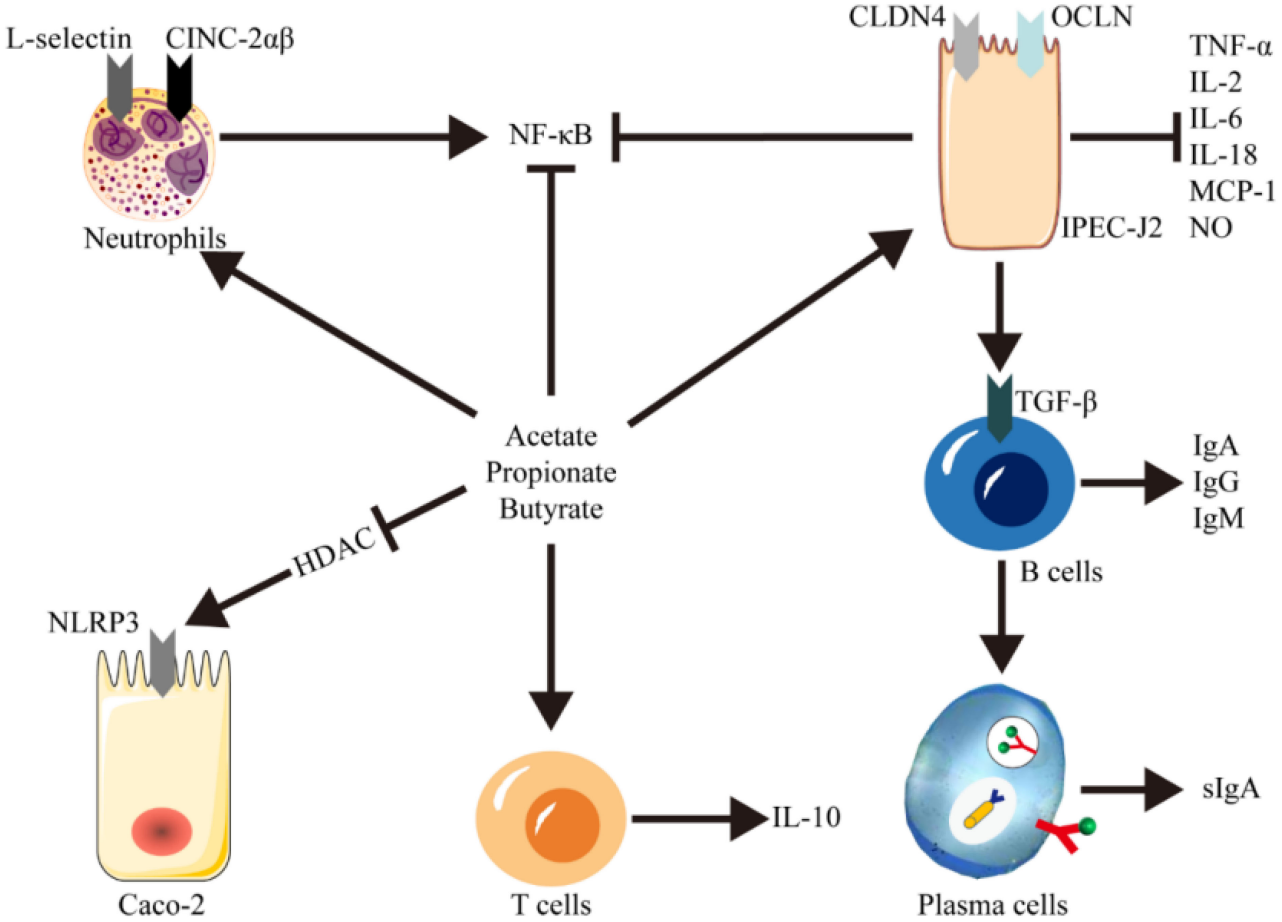
Immunomodulatory function of SCFAs on the intestinal immune system. SCFAs regulate local inflammation and protective immunity via activation of IgA, IgG, and IgM in B cells and promotion of differentiation into plasma cell sIgA via IPEC-J2 cells, enhancement of intestinal IL-10 expression via T cells, and promotion of neutrophil release and migration. Meanwhile, SCFAs-mediated HDAC inhibition, a crucial regulator of NF-κB activity and NLRP3 overexpression, lowered the expression of TNF-α, IL-6, IL-18 and MCP-1 in intestinal epithelial cells.

The activation of NLRP1 inflammasome leads to an increase in IL-18 production, which exacerbates dextran sulfate sodium (DSS)-induced colitis in C57BL/6 mice by reducing the abundance of butyrate-producing *Clostridium* species in the gut. However, the supplementation with 2% (mass to volume ratio) butyrate has been shown to mitigate the incidence of colitis in mice (Tye et al., 2018). Thus, appropriate concentrations of SCFA can suppress intestinal inflammation and enhance intestinal barrier function by inhibiting inflammasome activity. SCFAs function as histone deacetylase (HDAC) inhibitors, curbing the excessive activation of inflammasomes and thereby alleviating inflammation (Zou et al., 2022). Several studies have shown that treatment with specific concentrations of SCFAs, such as 0.5 mmol/L acetate, 0.01 mmol/L propionate, and 0.01 mmol/L butyrate, can inhibit HDAC expression and significantly suppress the overactivation of the NLRP3 inflammasome (Feng et al., 2018; Yuan et al., 2018; Wu et al., 2022). Furthermore, a recent study using a co-culture model of porcine intestinal epithelial cells (IPEC-J2) and peripheral blood mononuclear cells (PBMC) to simulate an acute inflammatory state has demonstrated that the addition of propionic acid can reduce the stimulated nitric oxide (NO) release from IPEC-J2 cells. Concurrently, it can induce an increase in the expression of tight junction protein (TJP) genes and promote the synthesis of Claudin-4 (CLDN4) and Occludin (OCLN) proteins (Andrani et al., 2023).This study highlights the protective effect of propionic acid against acute inflammation (**Figure 2**).

## 6 Conclusions and perspectives

A comprehensive review and analysis of existing literature indicate that short-chain fatty acids (SCFAs) exert a substantial influence on the immune system, intestinal health, and growth performance in pigs. Their effects encompass the preservation of intestinal mucosal barrier function, modulation of immune cell activity, regulation of inflammatory responses, and other mechanisms. Moving forward, research should delve deeper into the precise mechanisms through which SCFAs influence intestinal immunity and health in pigs, with a focus on microbiota-immune system interactions, immune tolerance, and the regulation of inflammation. Although SCFAs have been studied in the context of pig production, there is a need for more systematic and detailed investigations into their effects on pigs at various stages of growth. The response to SCFAs may vary significantly from the finishing stage to the lactation stage, highlighting the need for more targeted and ongoing research to offer more precise guidance for agricultural practices. While SCFAs are recognized for their benefits to intestinal health and the immune system, some studies have raised concerns about their potential to exacerbate inflammation and contribute to inflammation-related intestinal diseases. Thus, future research should aim to examine the biological effects of SCFAs in a more nuanced manner and explore possible solutions and preventative measures to mitigate any adverse effects.

This article makes a significant contribution by providing a thorough overview of the effects of short-chain fatty acids (SCFAs) in pig production. It delves into the multifaceted roles of SCFAs, including their influence on mucosal immune function, the modulation of adaptive immune responses, and their contribution to milk immune protection. By doing so, this article underscores the critical importance of SCFAs in maintaining and enhancing the health and productivity of pigs. Furthermore, the article illuminates the path for future research, emphasizing the need for ongoing studies to uncover additional insights into the mechanisms of action of SCFAs. It advocates for research that will yield scientific evidence to inform pig production practices and strategies for disease prevention. This forward-looking perspective is vital for the continued advancement of the field. The findings presented here carry substantial practical implications. They suggest that by optimizing the use of SCFAs, pig managers can potentially enhance the health and resistance of their animals. This optimization could lead to improved production performance, which is of paramount importance for the pig farming industry. Our review serves as a valuable resource for both researchers and practitioners alike, offering a synthesis of current knowledge and a roadmap for future exploration in the realm of pig health and production.
